# Setting the record straight on measuring SDG 4.2.1

**DOI:** 10.1016/S2214-109X(21)00256-4

**Published:** 2021-07-01

**Authors:** Bolajoko O Olusanya, Mijna Hadders-Algra, Cecilia Breinbauer, Andrew N Williams, Charles R J Newton, Adrian C Davis

**Affiliations:** Centre for Healthy Start Initiative, Lagos, Nigeria; University of Groningen, University Medical Center Groningen, Division of Developmental Neurology, Department of Paediatrics, Groningen, Netherlands; Center for Healthy Development, Seattle, WA, USA; Virtual Academic Unit, Northampton General Hospital, Northampton, UK; KEMRI-Wellcome Trust Research Program, Centre for Geographic Medicine Research (Coast), Kilifi, Kenya; Population Health Science, London School of Economics, London, UK

## Authors’ reply

We appreciate the attention of Mark Hereward from UNICEF to the concerns in our Comment published in *The Lancet Global Health*,^1^ and wish to clarify some of the alleged omissions and errors. We, and members of the public, are not privy to the internal processes and procedures that have restricted the development of a tool that includes all children younger than 5 years as required by Sustainable Develop Goal (SDG) 4.2.1, and that the component relating to children younger than 24 months is now the sole responsibility of WHO.

However, we wish to reaffirm the following facts that have not been refuted by UNICEF. First, as the sole custodian agency, UNICEF is fully accountable for SDG indicator 4.2.1.^2^ Second, the development of a survey tool for child functioning from birth was commissioned by the UN in 2001 and no appropriate tool has so far been developed by UNICEF for children younger than 24 months.^3^ Third, no indication exists as to when the required tool will be available for use. Fourth, SDG 4.2.1 has now been revised to exclude children younger than 24 months.^2^ Fifth, no published evidence exists of any systematic effort to review all the available population-level survey tools before the monumental decision to amend the SDGs to exclude children younger than 24 months.

To suggest that identification of children younger than 24 months with developmental delays is not possible in a survey tool or that individual-level tools and population-level tools are mutually exclusive is misleading. A widely embraced notion in global health, except for communicable diseases, is that where data are not available, there is no problem to warrant policy intervention. However, population-level data drive policy development and political support for investments in individual-level systems and tools for the early identification of children with developmental delays and disabilities. For instance, Chile has implemented an exemplary national early childhood development programme that is underpinned by successful developmental household surveys of children younger than 5 years since 2006.^4^

Additionally, the impression that the revised SDG 4.2.1 has been endorsed by the International Disability Alliance is erroneous. The joint statement issued in 2017 only referred to the disaggregation of SDG data by disability for all children and no reference whatsoever was made to children younger than 24 months.^5^

We are still deeply concerned about the collateral damage and far-reaching adverse consequences of the revised SDG 4.2.1 in the 6th year of the SDG because most of the affected families reside in sub-Saharan Africa and south Asia. We share the view that responding to this challenge requires collective action and thus renew the call on UNICEF to use their extensive network of partners and experts to attend to this task as a priority.
